# Supplementary figures and images for: Evaluation of laser induced sarcomere micro-damage: Role of damage extent and location in cardiomyocytes
Source: PLoS One. 2021 Jun 4;16(6):e0252346. doi: 10.1371/journal.pone.0252346 (PMC8177425; doi:10.1371/journal.pone.0252346)

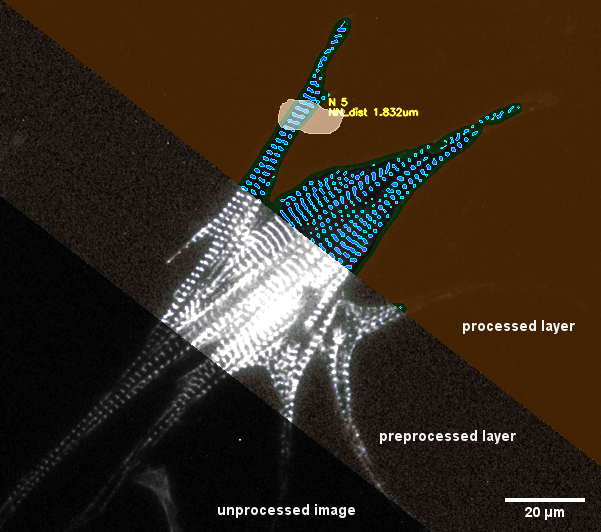

Supplement: S1 Fig — An OpenCV application was developed to quantify the total Z-disc count by single CMs. Therefore, unprocessed images were Fourier transformed, thresholded and via edge-detection refined to allow Z-disc counts in the processed layer. The representative image depicts the different processing layers and an example of a selected area and the resulting count of 5 Z-discs with a next-neighbor-distance of 1.83 μm. (TIF) [file pone.0252346.s001.tif]

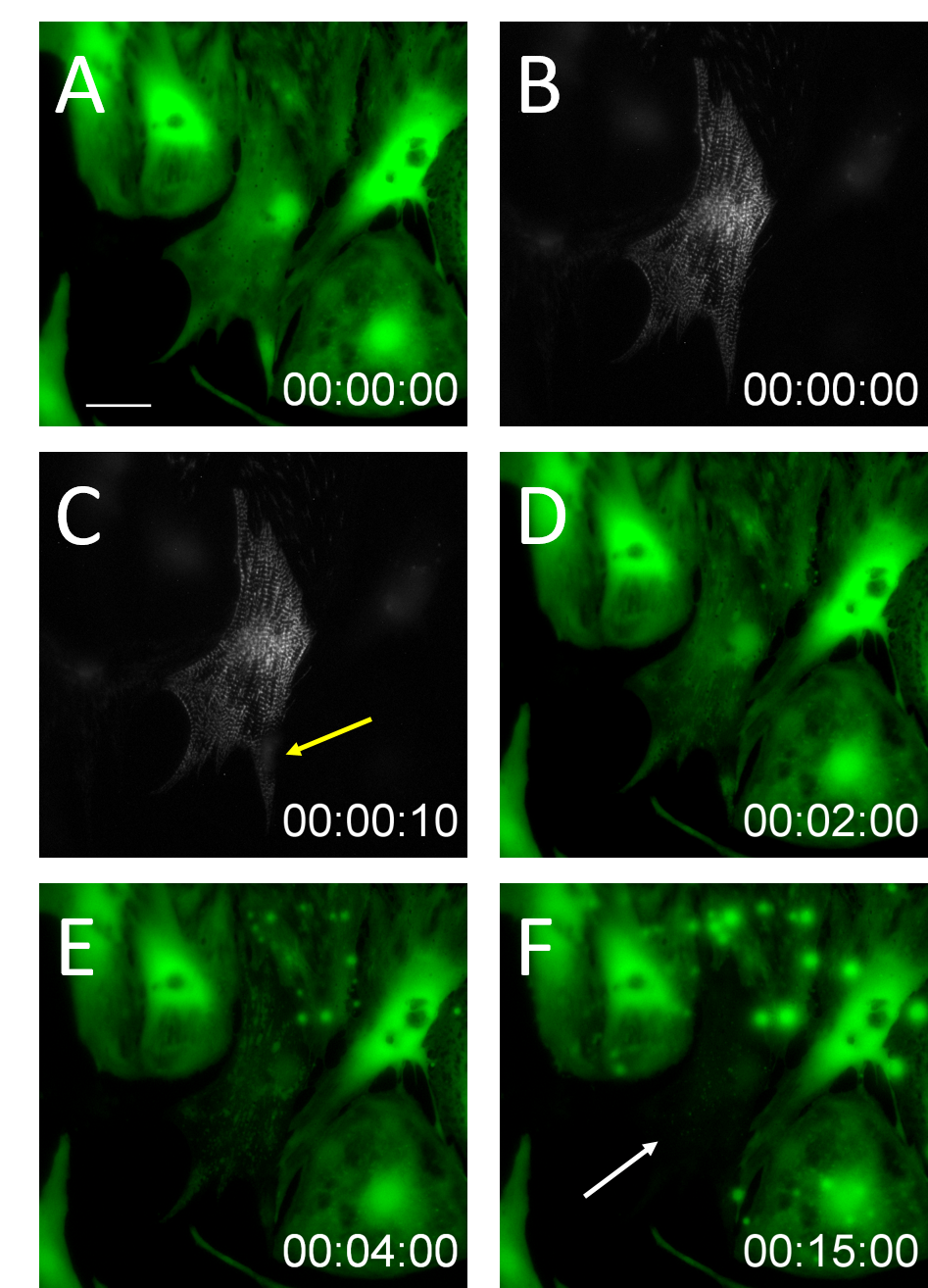

Supplement: S2 Fig — Using Calcein-AM dye, this representative image series depicts an active cell metabolism of a CM before (A) and a rapid cell death within minutes (D-F) after nanosurgery. A turboRFPlinked α-actinin expressing CM is shown before (A) and after ablating of 10 neighboring Z-discs (C, damage region indicated by the yellow arrow). 15 minutes post ablation (F), the treated CM did not show an active cell metabolism (white arrow). Accumulation of Calcein in untreated cells was found in all experimental trials after an extended incubation time with Calcein-AM. Scale bar 25 μm. (TIF) [file pone.0252346.s002.tif]

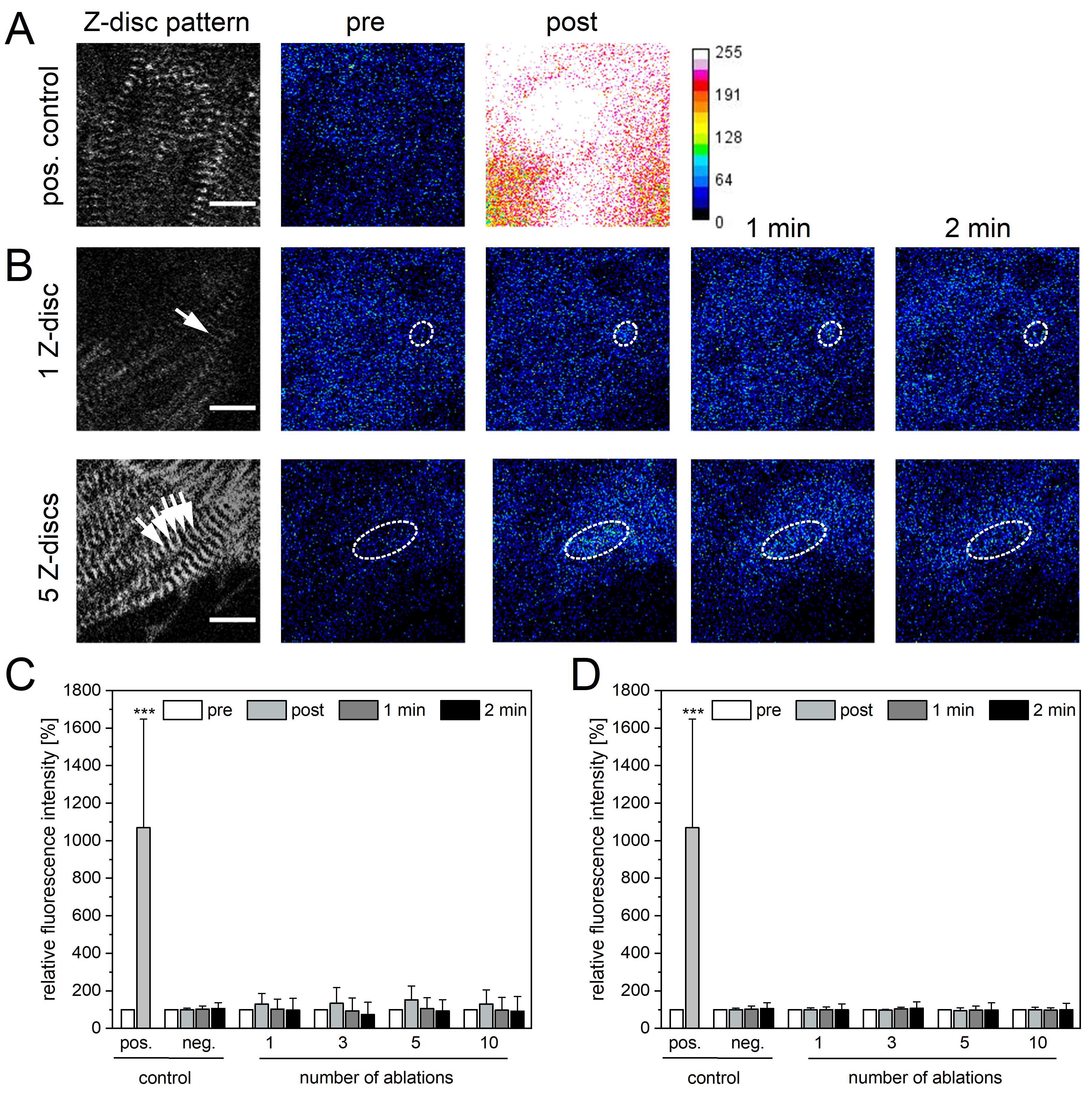

Supplement: S3 Fig — CMs were transduced with pLenti CMV mApple-ACTN2 Puro to visualize the Z-disc pattern and loaded with 10 μM 5-(and-6)-chloromethyl-2′,7′-dichlorodihydrofluorescein diacetate, acetyl ester (CM-H2DCFDA; Invitrogen, USA) for 30 min in DPBS with Ca2+ and Mg2+ to detect reactive oxygen species (ROS) after nanosurgery. After washing twice with Opti-MEM (Gibco 11058–021, Germany), Z-disc pattern and the basal fluorescence of H2DCFDA, at a wavelength of 850 nm, were recorded. Following ablation of different numbers of Z-discs, H2DCFDA fluorescence was assed directly, 1 min and 2 min post ablation. A representative time series for the ablation of one and five Z-discs (marked by arrows) and the region were H2DCFDA fluorescence was assed (dotted circle, pseudocolor) is shown in (B). H2DCFDA fluorescence was normalized to the pre ablation level and non-significant ROS generation was found after ablation of Z-discs in all treated cells (C) as well as in neighboring cells (D). A significant ROS increase was observed in the positive control, where 100 μM H2O2 was added (A). Untreated CMs served as negative control. Scale bars 10 μm. Bar charts represent relative fluorescence intensities (%) + standard deviation.***p < 0.01, One-Way ANOVA followed by Tukey test. ctr. n = 14, 1 Z-disc n = 20, 3 Z-discs n = 19, 5 Z-discs n = 14, 10 Z-discs n = 17. (TIF) [file pone.0252346.s003.tif]
