# Supplementary material for: Evaluation of laser induced sarcomere micro-damage: Role of damage extent and location in cardiomyocytes
Source: PLoS One. 2021 Jun 4;16(6):e0252346. doi: 10.1371/journal.pone.0252346 (PMC8177425; doi:10.1371/journal.pone.0252346)
Supplement: Attachment — Submitted filename: Revision - Response to Decision.pdf [file pone.0252346.s005.pdf]

Dominik Müller
Leibniz Universität Hannover
Institut für Quantenoptik
Welfengarten 1
30167 Hannover, Germany
+49 511 532 1364
d.mueller@iqo.uni-hannover.de

Response to Reviewers’ comments on the paper: **"Evaluation of laser induced sarcomere micro-damage: role of damage extent and location in cardiomyocytes"**

by Müller et al.

Article reference: PONE-D-21-00038

To the Editor

Dear Dr. Xu,

we gratefully appreciate the helpful comments and critique of the reviewers on our paper. We have addressed all points raised by the reviewers and thereby generated a revised and improved manuscript. Please find enclosed a detailed list of the changes in the manuscript and the respective answers to the comments of the reviewer.

Thank you for your time and consideration. We look forward to hearing from you in the near future.

Sincerely,

Dominik Müller

Points, answers and changes according to the first referee:

1. **Reviewers comment**: The authors note that 10 cell ablations do cause cellular death, but do not know the mechanism nor indicate that total power in the cell was controlled.

   Answer: Unraveling the mechanism of cellular death after ablation of single structures in CMs is challenging. In preliminary experiments, we observed an immediate cell death within minutes after ablation of 10 Z-discs (see S2 Fig). Therefore, we speculate that damage over a certain threshold (immediate cell death was not overserved after 5 Z-discs ablations) might result in disruption of the sarcolemma, as Z-discs are physically coupled to the sarcolemma via costameres[1]. A sufficient damage could result in sarcolemma instability or collapse and leaking of sarcoplasm. If so, this might be characteristic for a necrotic cell death[2]. In our earlier study form 2019[3], we detected in some CMs membrane blebbing (see Supplementary Figure S1.) after ablation of one Z-disc, which might indicate for an apoptosis-like pathway[2,4]. We want to unravel these cell death mechanisms and there dependency on the extent of damage with techniques like karyorrhexis detection, Annexin V staining and caspase activity measurements in further studies.

   In addition, we would like to note, that femtosecond laser based ablation is a non-linear process, which generates a low-density plasma in the focal region only (sub-femtoliter volume). This results in bond breaking of biomolecules without significant energy deposition (no heating). We refer to this process again below. It is in detailed reviewed by Vogel et al. 2005[5]. Due to the limitation to the focal region, the short lifetime of hydrated electrons or even formed ROS, the damage to adjacent regions, next to the targeted structure, is very unlikely.

   We have added experiments on ROS formation (see below) and a discussion on the possible cell death (see below and manuscript discussion and also 1st question of 2nd referee).

   a. **Reviewers comment**: This reviewer is concerned that the mechanism itself may be important to the interpretation of the author’s data. In both the earlier study and the current study, the authors do not study reactive oxygen species generation (although they clearly note it as a possible consequence in the 2019 study). The authors also do not shot show conclusively that Z-disc proteins are ablated, only that fluorescence is eliminated then re-formed. Thus, this reviewer is concerned that the authors are merely de-activating the fluorescence by causing the RFP or mCherry to ablate.
   b. Could the ablation itself and/or the laser input be causing ROS? If this is true, then the authors are studying local ROS generation instead of Z-disc dysfunction. Such a

mechanism may also provide a mechanism for cell death in the 10 ablation strategy. This reviewer suggests measuring the ROS production in the cells. If the authors cannot generate such data, this reviewer suggests that the authors provide a detailed comparison between their findings and findings in pathophysiological states that may alter Z-disc alignment, such as muscular dystrophy or nemaline myopathies.

Answer: a) In the mentioned earlier study from 2019, we performed dissection parameter studies to guarantee precise and confined ablation of Z-disc structures[3]. We evaluated different ablation parameters (wavelength, scanning velocity and laser power) on cell nuclei and used the most promising parameters to verify single Z-disc ablation via immunostaining against α-actinin (see Supplementary Figure S7 in Müller et al. 2019). In addition, we performed fluorescence recovery after photobleaching studies, to discriminate between bleaching of the Z-disc structure (see Supplementary Figure S2; half-recovery time 200s in Müller et al. 2019) and Z-disc reconstruction (typically >4h, see Figure 3C in Müller et al. 2019). We have clarified this in the revised manuscript.
Changes to the revised manuscript: "…these parameters were validated to result in Z-disc loss via subsequent immunostaining against α-actinin [11]."

Answer: b) Laser treatment of biological tissue can result in ROS mediated cell death, as seen e.g. for cytosolic irradiation with fs laser pulses[6]. However, the cellular response highly depends on the delivered energy. In the mentioned study from Yoon et al. 2015[6], cells were irradiated with energies of more than 1 µJ, which triggered ROS-mediated intracellular cell death pathways. In contrast, we used for our earlier and the present study ablation energies of 0.9 - 1 nJ. Nevertheless, we performed additional experiments to measure laser-induced ROS production in our setting. For this, we used CM-H_2DCFDA, which is a ROS indicator that emits green fluorescence when oxidized, to determine ROS formation after ablation of different numbers of Z-discs. We added our results as a separate figure to the supplements (S3 Fig). We observed negligible, non-significant local ROS generation after nanosurgery for all treated CMs (S3 Fig C) as well as for adjacent cells (S3 Fig D). Furthermore, fluorescence intensity 2 min after surgery was comparable with the pre-nanosurgery fluorescence level. Therefore, we speculate that CMs death, after ablation of Z-discs, is independent of ROS production.

c. **Reviewers comment:** While the authors note that the laser power settings were equalized for each experiment (Methods lines 151/152), they do not indicate that total power/energy delivery into the cell was normalized. This reviewer suggests: controlling for absolute power/energy delivered into the cell (for example 100 small

bursts of 10% of the ablation pulse through the cell) to determine if cell death is based the total power/energy delivered to the cell. Note that the reviewer believes that this may also provide evidence for or against the hypothesis that ROS production causes the cell death and damage.

Answer: Femtosecond laser-nanosugery produces a so-called low-density plasma of free electrons with negligible heat deposition using the mentioned laser parameters. This is different to longer pulsed laser systems, which also deposit heat/energy in biological specimen. A low-density plasma is characterized by ionization of biomolecules and water. The free electrons additionally produce bond breaking in biomolecules but do not diffuse out of the focal volume, due to their short half-life. The ablation process is dependent on the cellular region, for instance due to different densities of biomolecules. Therefore, it is difficult to speak of a total delivered energy. This is also in particular true as the energy deposition itself is very low as the thermal diffusion time is significantly longer than each laser pulse. This is also visible by the negligible ROS formation after Z-disc ablations (S3 Fig).

2. **Reviewers comment**: Discussion Paragraph around Line 422. The authors suggest that Refs 22, 29-31 indicate that myofibril disarray occurs. To this reviewer's reading, they refer primarily to cellular alignment (for example, Ref 31 discusses cellular myoFIBER alignment, not myoFIBRIL alignment. Reference 22 is an invitro model, not a true disease model. Only a very small statement in reference 29 about the pre-myofibril is clearly indicative of alignment. The authors should clearly note this limitation or provide stronger justification that such a small number of Z-discs would ultimately cause disease.

   Answer: We apologize for the misleading sentences and references. We have clarified our statement in the revised version of the manuscript and added appropriate references. "As myofibrillar rearrangement occurs during healthy, developing myocardium [29] but disarray after eccentric exercise [30] or in pathogenic phenotypes [31-33], we …" In addition, we added the following sentences to note the limitations of our results: "However, the main focus in this study is on the single cell level. Therefore, it is impossible to relate our findings to specific disease conditions."

3. **Reviewers comment:** Discussion Lines 456-457: The authors state that "This occurs probably by sarcomere reorganization under controlled contractility." The "probably" may be to strong as it is without evidence from this study, nor is it referenced.

Answer: We have attenuated our argumentation and added an appropriate reference. "This might be due to sarcomere reorganization during ongoing contraction [37]."

4. **Reviewers comment**: Results and Figures 2,4: The authors note that they did not observe a correlation between cell area and ablation. Similarly, they describe no clear correlation between distance from nucleus to ablation, except in the presence of an interaction with the number of sarcomeres. The legend of the figures are titled "Correlation of CMs…". However, Figures 2,4 only includes violin plots. Please provide statistical results regarding regression/correlations.

   Answer: We apologize for the misleading word „correlation" in the figure legends. We rephrased the word to relationship. Furthermore, text and figure legends now clearly indicate that we did not observed any significant relationship between cell area, number of Z-discs, location of damage and CMs viability. Violin plots were statically analyzed using a Kolmogorov-Smirnov test and p-values are now added in the revised manuscript

5. **Reviewers comment**: For figure 4, was an interaction between distance and number of nuclei included in the test? If not, could the authors include such or describe why it is not needed?

   Answer: Yes, we also included a statistical comparison between distance and number of nuclei in the test. First, we analyzed the relationship between damage distances to nucleus and viability of all treated CMs, independent of their number of nuclei (Fig 4B). From all treated CMs, we further discriminated between CMs with one nucleus (Fig 4C) and with two nuclei (Fig 4D) and analyzed the interaction between distance and viability. All data were tested for statistical significance with a Kolmogorov-Smirnov test. A statement that no significant differences were observed was added in the revised manuscript.

6. **Reviewers comment**: Results Lines 276-279: The authors report percent changes without statistics. Please clarify whether the data were significantly reduced by ablations or not.

   Answer: We corrected this sentence and included a significance statement: "A non-significant decreased viability from 68% (2 h) to 60% (24 h) was observed ($p = 0.99$) for the ablation of randomly selected Z-discs and from 81% (2 h) to 60% (24 h) in case of neighboring Z-discs ($p = 0.59$)."

7. **Reviewers comment**: Results Lines 369-371: Please indicate the reported power of this test to provide confidence that the reduced shortening is not a Type II (false positive). statistical error

   Answer: We corrected this statement and included the p-value and the statistical power: "However, the sarcomere shortening decreased significantly (p = 0.035, with a statistical power of 68%) from 7.7% (post ablation) to 4.7% (2 h post ablation) by ablation of a single Z-disc in CMs (Fig 7D)."

8. **Reviewers comment**: The authors note throughout that number and distance to the nucleus differed. However, they do not include a hypothesis or justification for looking at nuclei instead of other cellular compartments (for example the endoplasmic reticulum or mitochondria). Please describe why this and/or the finding that 2 nuclei improve survival is an important factor to study.

   Answer: The Z-disc is not only a structural and scaffolding complex but also participates in signal transduction events. For instance, nuclear translocation was reported for muscle LIM proteins (MLPs) in response to extrinsic pressure overload leading to MLP mediated cell-fate determination and tissue-specific gene expression[7,8] or anti-apoptotic effects were investigated after cGMP-dependent nuclear trafficking of zyxin from the Z-disc to the nucleus and the activation of Akt in cardiomyocytes[9]. Hence, we were interested if the distance between damage and nucleus implies a different consequence for CM survival. In the near future, we plan to address further cellular compartments and their role during endogenous regeneration and damage response in cardiomyocytes.

9. **Reviewers comment**: Results Lines 343-344: The authors describe that alignment within a 20 degree angle was compared. Please include a statement of why this range was chosen.

   Answer: We have chosen an angle of 20° to detect clear sarcomere disorganization. A smaller angle might be too sensitive and could lead to false positive results. To clarify this, we added a note into the revised version of the manuscript: "We also compared the angular distributions of myofibril alignment in the relaxed (Fig 6A) and contracted (Fig 6B) cell state of CMs. Therefore, the percentage of sarcomeres within a range of 20°, as a robust angle for disarray detection, was compared to the post ablation alignment."

10. **Reviewers comment**: Figure 6 Panels C,D: Please check the comparative text to see if the "pre" and "post" labels have been flipped. (E.g. the analysis was unlikely to be done 2 h PRE ablation).

Answer: We would like to thank the reviewer for pointing us to this mislabeling, which we have corrected in the revised version of the manuscript.

Points, answers and changes according to the second referee:

1. **Reviewers comment**: The manuscript by Mueller et al. entitled "Evaluation of laser induced sarcomere micro-damage…" is devoted to evaluation of method to introduce micro-damage in sarcomere. This new laser-based approach allows for the precise ablation of individual elements of the sarcomere, i.e. Z-disc. In their previous article (Mueller at al. 2019) the authors established the femtosecond laser-based system with the sub-micrometer precision cell manipulation. Here, they demonstrate correlation between spatially confined micro-damages and cell survival. The problem of the study is very important: many cardiomyopathies develop sarcomere disarray demonstrating cardiomyocyte losses. The femtosecond laser-based nanosurgery followed by the fluorescence microscopy is a powerful tool to better understand integrity and structure-function of the sarcomeric machinery in healthy and disease conditions. These evaluation assays might be of interest for the researchers who study cardiomyopathies: this is a good model of the sarcomere disarray.

   These methods will also help to investigate cytoskeleton structure-function and sarcomere reparation.

   I suppose that this interesting methodological research article can be published in PLoS One, just some flaws need to be corrected and some important questions should be addressed prior publication.

   Answer: The authors are grateful to this positive comment and the advice and support given in the following points of the review.

2. **Reviewers comment**: Authors should convince a reader that the ablation of the Z-disk really took place. Rat newborn cardiomyocytes were transfected with anti-a-actinin fused with turbo-RFP for visualization of Z-disks. The 'free-electron-mediated bond-breaking' can also affect structure of the RFP reporter, recognition site of antibody etc. Authors should show phase microscopy photographs in parallel to the fluorescence microscopy images for reader to ensure that the damage really took place in the Z-disk proteins.

Answer: The reviewer is right, that a clear ablation of Z-discs is crucial for all following analysis. Therefore, we performed several dissection parameter studies in our previous research article (see answer to first Referee 1a). Furthermore, we would like to mention, that transfection with RFP linked alpha-actinin results in expression of fusion alpha-actinin as well as concurrent expression of endogenous, native alpha-actinin. Hence, an affected RFP reporter would not hamper the native Z-disc pattern. To convince the reader further, we added a video sequence to the supplements and referred to the video in the revised manuscript: "a single Z-disc ablation could lead to a significantly different angular distribution (S1 Video)." In this video, a contracting Z-disc pattern before and post ablation of a single Z-disc is depicted. In the post ablation patter, a retraction of neighboring Z-discs is evident. Hence, this could not be achieved via photobleaching but rather is a sign of destruction. In addition, our SGFT data indicate that Z-disc ablations result in ≥ 40% of CMs to significantly different angular distributions on a single cell level. Which might not occur after photobleaching events. We think that phase contrast measurements are a promising method to confirm Z-disc damage for isolated myofibrils, but are less suited to prove ablation in a dense cytoskeletal network of living CMs. We tried to image the Z-disc using holographic tomography (Nanolive 3D explorer), which has a very high sensitivity for quantitative phase imaging. However, the Z-disc pattern could not be revealed. Another possibility to reveal Z-disc ablation would have been to use electron microscopy; however, this would demand successful rediscovery of the treated cell.

3. **Reviewers comment**: How cell area was measured? It is not clear from the Methods. For the in vivo cardiomyocyte membrane visualization, I would recommend di-4 ANEPPS membrane voltage -sensitive dye.

   Answer: The cell area was measured by circling the visualized cytoskeleton, comparable to the earlier study from 2019, Figure 4. In this previous study, we had validated this method. We added a comment into the revised version of the manuscript. "Furthermore, the visualized cytoskeleton of CMs was encircled with Fiji to calculate the ratio of cell area to Z-discs per cell."

4. **Reviewers comment**: What was the cause of the cell death after ablation(s)? This important question should be addressed in Discussion. If the main purpose of the paper is evaluation of the method, and cell viability is most important parameter, this question at least should be discussed.

Answer: We discussed the possible cell death mechanisms in more detail in the answer to the first reviewer (1. reviewer comment) and added an appropriate section to the revised manuscript.

"Increased cytoskeletal damage results in increased mortality of CMs.

However, the damage response of CMs is multiphasic and cell death mechanism might depend on the damage extent. In our earlier study, we detected membrane blebbing in some of the treated CMs after ablation of a single Z-disc, which might indicate an apoptosis-like pathway [24,25]. In the present study, we observed an immediate cell death within minutes after ablation of 10 Z-discs without membrane blebbing. Therefore, it might be that damage over a certain threshold lead to disruption of the sarcolemma, as Z-discs are physically coupled to the sarcolemma via costameres [26]. Studies have shown that the sarcolemma of contracting cells is very susceptible to damage [24,27]. Thus, sufficient damage could result in sarcolemma instability or collapse and leaking of sarcoplasm. If so, this might be characteristic for a necrotic cell death pathway [24]. Nevertheless, this hypothesis needs to be evaluated in further studies. An induced cell death via reactive oxygen species (ROS) is unlikely, as we could not detect significantly elevated ROS formation after laser-induced Z-disc ablations (S3 Fig)

5. **Reviewers comment**: Line 90: 'influence of location of the damage'. Influence on what?

   Answer: The reviewer is right that the statement to the damage location is misleading. We added a comment into the revised version of the manuscript. "Secondly, we investigated if the location of the damage is associated with cell viability"

6. **Reviewers comment**: Line 233: add words 'within 2 hrs after damage' Do the same for the Figure 1 capture. Why did authors choose 2 hrs interval in those experiments? Why in some experiments authors use 24 hrs instead of 2 hrs?

   Answer: We added the 'within 2 h after damage' phrase in Line 235 and Line 250. In preliminary experiments, we observed cell death of CMs after ablation of 10 Z-discs within minutes (S2 Fig). Hence, we were interested, if such an early cell death is also detectable for less than 10 Z-discs ablations. Since we observed a relative high survival rate for 2 h after ablation in the case of 3 Z-discs, we wanted to see if the viability changes with time. We found a non-significant reduction for 3 ablations and expected the same for less than 3 Z-discs ablations, as also indicated by our previous study. Thus, we measured the viability in following experiments after 24 h. For SGFT and SarcTrack experiments, the focus in this study was to decipher the early sarcomere organization, why we choose the immediate and 2 h post ablation time points.

7. **Reviewers comment**: Figure 1. It might be a good idea to show a correlation plot Viability vs # of Z-disk ablations.

   Answer: Thank you for the suggestion of a correlation plot. On purpose, we decided not to perform a correlative or regression analysis of the pure viability data. To plot a correlation, it is crucial to have a linear association between the viability and the number of ablations. We expected a nonlinear relationship, for instance by more than >> 10 Z-discs ablations all CMs will die, which is difficult to represent and could be misleading.

8. **Reviewers comment**: Line256: "A critical factor for CMs survival […] could be the size and the total number of Z-disks…" This is not good hypothesis to test. Hypothesis should be something like "CMs with smaller cell area more susceptible to the damage "

   Answer: We agree with the reviewer comment and rephrased the hypothesis as follows: "Due to the strong interconnection of Z-discs in CMs, we hypothesized that smaller CMs and CMs with few Z-discs are more susceptible to Z-disc ablation, compared to large CMs or CMs with many Z-discs."

9. **Reviewers comment**: Line 263: 'Cardiomyocytes with fewer Z-disc counts…' Very confusing sentence. Need to re-phrase.

   Answer: We agree with the reviewer. We rephrased the sentence in the revised manuscript. "Cardiomyocytes with a low number of Z-discs tended to survive the ablation of 5 Z-discs better compared to those with more Z-discs."

10. **Reviewers comment**: Line 277 and 279: Were these differences significant?

    Answer: The differences were not significant and the p-values are now mentioned in the revised manuscript (see also comment 6 of the first referee).

11. **Reviewers comment**: Figure 4, panels C and D: Are these differences significant? How to treat these violin plots w/o statistical significance?

    Answer: We decided to plot the raw data as violin plots to outline changes in the distribution of the data. To have a statistical comparison, a Kolmogorov-Smirnov test was performed. No significant differences were observed. This statement is added in the

figure 4 caption and in the text of the revised manuscript. Please refer to line 308-309, 315 and 321-322.

12. **Reviewers comment**: Line 308: Why opposite correlation was observed in CMs with two nuclei? Significant?

    Answer: The correlation was not significant ($p \geq 0.48$) and this is now added in the revised manuscript.

13. **Reviewers comment**: Figure 5. Mistype: pattern strengTH. Could authors explain for a reader how to read these pattern strength maps? It is not easy.

    Answer: We corrected the error in Figure 5 and added a note to the caption to better explain the pattern strength maps: "The pattern strength heat map visualized regions with high levels of repeating patterning in yellow/white and less repeating structures in darker red."

14. **Reviewers comment**: Line 317: What is 'continuous' contractility? Contractility is a property of CMs: it can't be continuous. Contraction?

    Answer: We agree with the reviewer. Continues contractility was replaced with contraction.

15. **Reviewers comment**: Line 330: It is not clear where. Authors should point out the location where 5 Z-disks were ablated.

    Answer: The reviewer is right. We implemented a yellow arrow in Figure 5 to point to the location were 5 Z-discs were ablated. We also corrected the sentence to: "… clearly indicates the ablation of 5 Z-discs (Fig 5B, yellow arrow)." and added a comment to the caption of Figure 5.

16. **Reviewers comment**: Figure 6, panels C and D: note the units (% of cells)

    Answer: We corrected the Figure 6 and added the units.

17. **Reviewers comment**: Figure 7, panel D: why only single Z-disk damage reflected in sarcomere shortening? It is not convincing for a reader.

Answer: We fully agree with the reviewer. We were also surprised about this finding. We speculate that ablation cause minor time-dependent retraction of adjacent sarcomeres, leading to reduced sarcomere shortening rates. Furthermore, it might be that endogenous repair happens faster after single Z-disc ablation compared to more Z-discs ablations. A possible explanation could be a temporal stabilization of missing Z-disc elements (e.g. via FilaminC)[10], resulting in reduced sarcomere shortening. We are currently planning further experiments in this direction, including immunostaining studies to detect supporting structure development and their influence on sarcomere assembly. We added a statement in the revised manuscript: "The significant reduction of sarcomere shortening in case of ablation of a single Z-disc might speculatively be related to faster endogenous repair. However, this needs to be analyzed in future experiments."

18. **Reviewers comment**: Line 387: Did authors study sarcomeregenesis?

    Answer: In the current manuscript, we did not specifically study sarcomergenesis. Therefore, we removed the "… as an anchoring complex during sarcomerogenesis …" statement in the revised manuscript. In our previous publication (Müller et al. 2019) some measurements on the repair rate of the sarcomere over time were performed. We aim to extend this analysis on a molecular biological level in future experiments to determine if Z-disc pattern repair is based on sarcomerogenesis.

19. **Reviewers comment**: Line 397-398: to make this conclusion authors needed to compare randomly to neighboring Z-disks damage. This comparison had not been made.

    Answer: The reviewer is right that our statement was misleading. Therefore, we deleted the section: "Interestingly, the viability was more affected by introducing the damage randomly compared to ablation of neighboring Z-discs. Therefore, we speculate that localized damage can be more intercepted than multiple allocated damage." in the revised manuscript.

20. **Reviewers comment**: Lines 443-447. Forces can't be directly quantified from these experiments.

    Answer: The reviewer is right that we cannot directly quantify force generation via sarcomere length measurements. Therefore, we changed our statement as follows: "While maximal force is generated close to resting sarcomere length [36], it might be that force generation is not significantly affected after Z-disc ablations."

References

1. Peter, A. K., Cheng, H., Ross, R. S., Knowlton, K. U. & Chen, J. The costamere bridges sarcomeres to the sarcolemma in striated muscle. *Prog. Pediatr. Cardiol.* **31**, 83–88 (2011).
2. Kung, G., Konstantinidis, K. & Kitsis, R. N. Programmed necrosis, not apoptosis, in the heart. *Circ. Res.* **108**, 1017–1036 (2011).
3. Müller, D. *et al.* Femtosecond laser-based nanosurgery reveals the endogenous regeneration of single Z-discs including physiological consequences for cardiomyocytes. *Sci. Rep.* **9**, 3625 (2019).
4. Galluzzi, L. *et al.* Molecular mechanisms of cell death: Recommendations of the Nomenclature Committee on Cell Death 2018. *Cell Death Differ.* **25**, 486–541 (2018).
5. Vogel, A., Noack, J., Hüttman, G. & Paltauf, G. Mechanisms of femtosecond laser nanosurgery of cells and tissues. *Appl. Phys. B Lasers Opt.* **81**, 1015–1047 (2005).
6. Yoon, J., Ryu, S. W., Lee, S. & Choi, C. Cytosolic Irradiation of Femtosecond Laser Induces Mitochondria-dependent Apoptosis-like Cell Death via Intrinsic Reactive Oxygen Cascades. *Sci. Rep.* **5**, (2015).
7. Arber, S., Halder, G. & Caroni, P. Muscle LIM protein, a novel essential regulator of myogenesis, promotes myogenic differentiation. *Cell* **79**, 221–231 (1994).
8. Ecarnot-Laubriet, A. *et al.* Downregulation and nuclear relocation of MLP during the progression of right ventricular hypertrophy induced by chronic pressure overload. *J. Mol. Cell. Cardiol.* **32**, 2385–2395 (2000).
9. Kato, T. *et al.* Atrial natriuretic peptide promotes cardiomyocyte survival by cGMP-dependent nuclear accumulation of zyxin and Akt. *J. Clin. Invest.* **115**, (2005).
10. Leber, Y. *et al.* Filamin C is a highly dynamic protein associated with fast repair of myofibrillar microdamage. *Hum. Mol. Genet.* **25**, 2776–2788 (2016).

We again like to thank the editor and reviewers for their help and valuable comments. We hope that we have sufficiently dealt with all addressed points.
