# Supplementary material for: Evaluation of laser induced sarcomere micro-damage: Role of damage extent and location in cardiomyocytes
Source: PLoS One. 2021 Jun 4;16(6):e0252346. doi: 10.1371/journal.pone.0252346 (PMC8177425; doi:10.1371/journal.pone.0252346)
Supplement: Attachment — Submitted filename: Response to Reviewers.docx [file pone.0252346.s006.docx]

Dominik Müller

Leibniz Universität Hannover

Institut für Quantenoptik

Welfengarten 1

30167 Hannover, Germany

+49 511 532 1364

d.mueller@iqo.uni-hannover.de

Response to Reviewers’ comments on the paper: **"Evaluation of laser induced sarcomere micro-damage: role of damage extent and location in cardiomyocytes"**

by Müller et al.

Article reference: PONE-D-21-00038R1

To the Editor

Dear Dr. Xu,

we are grateful for the constructive comments and critique of the reviewers on our revised paper. We have addressed all points raised by the reviewers and thereby generated a revised and improved manuscript. Please find enclosed an unmarked version of the revised manuscript, a manuscript with highlighted changes and the respective answers to the comments of the reviewers.

Thank you for your time and consideration. We look forward to hearing from you in the near future.

Sincerely,

Dominik Müller

***Points, answers and changes according to the first reviewer:***

1. **Reviewers comment**: The submission PONE-D-21-00038R1 by Muller et al, argues that ablation of multiple z-disks using a femtosecond laser is associated with cell death and damage to contractile function, related to mofibril disarray. While the authors have substantial evidence to suggest that the laser treatment causes z-disc misalignment, this reviewer remains unconvinced that the cell death is due to z-disc ablation and not the laser treatment. Nonetheless, the authors report unique methodology and provide improved context and limitations within the discussion.

**Answer:** We thank the reviewer for his constructive comments, which we have used to improve the presentation of our data/statistics and to include another statement on the cell death. As we detail below, it is very difficult to determine an ultimate cause of cell death, due to methodological constraints. A comparison to other cells is not doable, because in our experience nanosurgery highly depends on cell type, line, differentiation state, staining and further factors. The only option to get a good understanding on the final mechanism would be RNA Sequencing. However, as the amount of cells which can be laser treated is limited, this also limits highly the amount of mRNA and currently we have not an easy solution to address this limitation. Please see below for more detailed comments.

2. **Reviewers comment**: While this reviewer is grateful that the authors improved their reporting of statistical results, the revised results section highlights several concerns. For example, line 281-282 of the highlighted manuscript states "A non
   significant decreased viability from 68% (2 h) to 60% (24 h) was observed (p = 0.99)...". It is unclear why the authors would highlight changed percentages when the differences were not statistically significant. Even for data nearing p~0.15, it would seem that the authors are purposely highlighting a difference that is not there. Given the sample sizes, this reviewer would suggest removing statements that state differences or trends in mean differences when the data are not near p=0.05.

**Answer:** This point was also raised by the 2^nd^ reviewer. Please excuse that we have not been more careful when we prepared the 1st revision and had removed these points. We fully agree with the reviewers and have now removed the misleading sentences (Lines 246-248, 265-270, 281-284, 305-309, 310-315, 433-437) in the revised manuscript.

The following sentences were changed (former version/revised version):

Line 246-248

“A non-significantly decreased viability was observed after ablation of 3 Z-discs, with a higher survival rate after ablation of neighboring Z-discs in a set (81%) compared to randomly ablated Z-discs (68%).” 🡪 “A non-significantly decreased viability was observed after ablation of 3 Z-discs.”

Line 265-270

“The survival was also independent of the total Z-disc count (p = 0.09) within the cell. We found a similar distribution after ablation of 3 Z-discs for both ablation patterns. Cardiomyocytes with a low number of Z-discs tended to survive the ablation of 5 Z-discs better compared to those with more Z-discs. In contrast, the opposite effect was observed for 10 Z-discs ablations.” 🡪 “We also counted the Z-discs within the cell area and could not detect a significant influence of the Z-disc count (p ≥ 0.09) on the cell survival.”

Line 281-284

„A non-significant decreased viability from 68% (2 h) to 60% (24 h) was observed (p = 0.99) for the ablation of randomly selected Z-discs and from 81% (2 h) to 60% (24 h) in case of neighboring Z-discs (p = 0.59).” 🡪 “A non-significant decreased viability was observed for the ablation of randomly selected Z-discs (p = 0.99) as well as for the ablation of neighboring Z-discs (p = 0.59).”

Line 305-309

“Furthermore, the micro-damage distance to the nucleus was not significantly influencing the viability of CMs (Fig 4B). A slight trend to reduced viability is visible after Z-disc ablation in the range of 20 to 45 µm away from the nucleus. However, a direct statistical comparison of pre and post ablation groups revealed no statistically significant differences (p = 0.58).” 🡪 “Furthermore, the micro-damage distance to the nucleus was not significantly influencing the viability of CMs (p = 0.58, Fig 4B).”

Line 310-315

“We also assessed whether the number of nuclei and the damage distance to the nucleus are related to cell survival. The data indicated a higher cell survival rate in CMs with one nucleus and ablation of Z-discs close to the nucleus (Fig 4C). In CMs with two nuclei, the distance to the ablated Z-disc was determined to the nearest nucleus. The opposite association with a lower survival rate was detected in CMs with two nuclei (Fig 4D). However, these were not statistically different (p ≥ 0.48).” 🡪 “We also assessed whether the number of nuclei and the damage distance to the nucleus are related to cell survival (Fig 4C, 4D). In CMs with two nuclei, the distance to the ablated Z-disc was determined to the nearest nucleus. No statistically different viabilities were observed (p ≥ 0.48).”

Line 433-437

“By comparing the viability pre and 24 h post ablation of a single Z-disc per CM, the median distributions were similar. Furthermore, we observed a higher cell survival rate by CMs with one nucleus and ablation of Z-discs close to the nucleus. The opposite dependency was detected by CMs with two nuclei. In earlier studies …” 🡪 “By comparing the viability pre and 24 h post ablation of a single Z-disc per CM, the median distributions were similar. In earlier studies …”

3. **Reviewers comment:** Statistics: Given that Viability (Figure 1C/D) included two factors (control/treated; number of ablations), why are the authors reporting a one-way ANOVA instead of a multi-factor ANOVA?

**Answer:** Each control group represents non-treated CMs (0 Z-disc ablations) within the same wells as treated ones, meaning each experimental condition (10, 5 or 3 Z-disc ablations) has its own control. Therefore, for this experiment, a one-way ANOVA was used to compare non-treated with Z-disc ablated CMs, independently of the amount of Z-disc ablations. We clarified this in the Fig 1 caption: “Treated CMs were statistically compared with untreated, control CMs (individual determined for each experiment) using a One-Way ANOVA followed by Tukey test (**p < 0.01, and *p < 0.05).”

4. **Reviewers comment**: (Related to Reviewer's original Comment 1) While this reviewer understand the author's interest in further pursing the mechanisms of cell death in future studies (and greatly appreciates the inclusion of the new ROS data and expanded discussion), this reviewer is not convinced that the z-disc removal causes the cell death. This reviewer would be more comfortable with the results if they showed that femtosecond laser treatment was not the cause of cell death. (For example, via distributed treatment, as previously suggested, or by treating a non-sarcomeric structure with the same ablation (either in a muscle cell away from nuclei and striations, which is difficult, or a non-muscle cell type).

**Answer:** We acknowledge that the reviewer is not fully convinced that the loss of Z-discs is the ultimate cause of cell death. We agree that potential downstream effects of femtosecond laser treatment, in particular after treatment of many Z-discs, might also influence cell survival. We added a statement to the text to emphasize that we cannot fully exclude further laser-related mechanisms of cell death: “Nonetheless, it is possible that the application of the nanosurgery itself will induce further downstream effects, which influence cell survival, in particular, for many Z-disc ablations. However, this cannot be reliably elucidated due to the strong dependence of fs laser-based nanosurgery on the cell type, state and the ablated structure [12].”

The ultimate cause of cell death cannot be analyzed in a comprehensive way. As already point out by reviewer 1, the application of similar damage (comparable to ablation of multiple Z-discs) to non-sarcomeric elements within a cardiomyocyte is extremely challenging due to space constraints. However, also an extrapolation or comparison of CMs to other cell types is not possible. These present different cytoskeletal architectures, different expression patterns and mechanical connections between cytoskeletal elements. Based on our experience, the threshold for successful ablation varies a lot between biological specimen and after staining. We have recently shown, that laser parameters must be chosen carefully, depending on the staining of the cell, the cell’s (differentiation) state, and the extent of the cut region, such that unwanted cell responses can be avoided (Hagenah et al. 2018)^1^. Even on a subcellular level, different organelles require different ablation power. Therefore, comparing the effect of fs laser-based ablation in CMs to other cells or structures is nearly impossible. We think that single cell sequencing could be a way to elucidate cellular reaction after laser treatment on a molecular level. To address this, we have started to establish a technique to analyze the expression profile of distinct, single CMs after treatment. However, this is still at a very early stage due to large challenges concerning the amount of RNA.

***Points, answers and changes according to the second reviewer:***

1. **Reviewers comment**: The manuscript by Mueller et al. entitled “Evaluation of laser induced sarcomere micro-damage…” was significantly improved in my opinion.

**Answer:** The authors are grateful to this positive comment and the advice given in the following points.

2. **Reviewers comment**: I still do not like lines 259-261. Authors should mention that this is amount of Z-disks in ROI, not total. I would stick to the parameter ‘cell area’ instead of amount of Z-disks. I think that is most suitable.

**Answer:** We revised our statement to clarify that the amount of Z-discs within the cell area was counted. We also rephrased the paragraph to put emphasize to the “area” parameter. “Due to the strong interconnection of Z-discs in CMs, we hypothesized that smaller CMs are more susceptible to Z-disc ablation, compared to large CMs. To examine this hypothesis, the cell area of all CMs was analyzed before and after the ablation of multiple Z-discs (Fig 2). We did not observe a significant relationship between cell area and CM survival, neither for ablation of Z-discs in a set (p ≥ 0.14) nor for randomly selected Z-discs (p ≥ 0.81) within the cell. We also counted the Z-discs within the cell area and could not detect a significant influence of the Z-disc count (p ≥ 0.09) on the cell survival.”

3. **Reviewers comment**: I would suggest not to make ANY conclusion on insignificant data (Lines 268-269; also lines 314-315). Please, check throughout whole manuscript.

**Answer:** We removed conclusions about insignificant data (Lines 246-248, 265-270, 281-284, 305-309, 310-315, 433-437) in the revised version of the manuscript (please also refer to the first comment of reviewer 1).

Reference:

1.	Hagenah, D., Heisterkamp, A. & Kalies, S. Effects of cell state and staining on femtosecond laser nanosurgery. *Journal of Biophotonics* e201700344 (2018) doi:10.1002/jbio.201700344.

*We again like to thank the editor and reviewers for their help and valuable comments. We hope that we have sufficiently dealt with all addressed points.*
