# Supplementary material for: Evaluation of laser induced sarcomere micro-damage: Role of damage extent and location in cardiomyocytes
Source: PLoS One. 2021 Jun 4;16(6):e0252346. doi: 10.1371/journal.pone.0252346 (PMC8177425; doi:10.1371/journal.pone.0252346)
Supplement: Attachment — Submitted filename: Response to Reviewers.docx [file pone.0252346.s007.docx]

Dominik Müller

Leibniz Universität Hannover

Institut für Quantenoptik

Welfengarten 1

30167 Hannover, Germany

+49 511 532 1364

d.mueller@iqo.uni-hannover.de

Response to Reviewers’ comments on the paper: **"Evaluation of laser induced sarcomere micro-damage: role of damage extent and location in cardiomyocytes"**

by Müller et al.

Article reference: PONE-D-21-00038R2

To the Editor

Dear Dr. Xu,

we are grateful for the constructive comments and critique of the reviewers on our revised paper. We have addressed the point raised by the reviewers and thereby generated a revised and improved manuscript. Please find enclosed an unmarked version of the revised manuscript, a manuscript with highlighted changes and the respective answers to the comments of the reviewers.

Thank you for your time and consideration. We look forward to hearing from you in the near future.

Sincerely,

Dominik Müller

***Points, answers and changes according to the first reviewer:***

1. **Reviewers comment**: The authors, Muller et al, further revise PONE-D-21-00038R2, "Evaluation of laser induced sarcomere micro-damage: role of damage extent and location in cardiomyocytes". The authors provide data suggesting that z-disc ablation using a femtosecond laser is associated with both cell death and myofibril re-alignment.

The authors have addressed the most substantial concerns, but this reviewer suggests further edits on statistical reporting for one result: Line 278/279 (results for figure 3). It does not make sense to this reviewer that a p=0.99 and 0.59 be highlighted as "a non-significant decreased viability". Since the graphs do visually show stronger trends, this reviewer suggest re-evaluating the data used to derive this statistic. If the p-values are replicated, this reviewer suggest that the authors highlight that 'despite a visual difference' there was no statistically significant difference in viability, instead of maintaining the wording in the current draft.

**Answer:** We re-evaluated and confirmed the p-values form figure 3 and changed our statement as follows:

The metabolic activity was visualized 2 h or 24 h after Z-disc ablation with Calcein-AM (Fig 3). No statistically significant differences in viability were observed for the ablation of randomly selected Z-discs (p = 0.99) as well as for the ablation of neighboring Z-discs (p = 0.59).

***Points, answers and changes according to the second reviewer:***

1. **Reviewers comment**: I am satisfied how the reviewer's questions were addressed by the authors. I think the manuscript deserved to be published in PLoS One

**Answer:** We appreciate the positive feedback.

*We again like to thank the editor and reviewers for their help and valuable comments. We hope that we have sufficiently dealt with the addressed point.*
